# Neurocognitive Impairment in ART-Experienced People Living with HIV: An Analysis of Clinical Risk Factors, Injection Drug Use, and the sCD163

**DOI:** 10.3390/v17091232

**Published:** 2025-09-10

**Authors:** Syed Zaryab Ahmed, Faiq Amin, Nida Farooqui, Zhannur Omarova, Syed Faisal Mahmood, Qurat ul ain Khan, Haider A. Naqvi, Aida Mumtaz, Saeeda Baig, Muhammad Rehan Khan, Sharaf A. Shah, Ali Hassan, Srinivasa Bolla, Shamim Mushtaq, Syed Hani Abidi

**Affiliations:** 1 Department of Biochemistry, Ziauddin University, Karachi 75000, Pakistan; syed.zaryab@zu.edu.pk (S.Z.A.); saeeda.baig@zu.edu.pk (S.B.); 2 Department of Biological and Biomedical Sciences, Aga Khan University, Karachi 74800, Pakistan; faiq.amin@aku.edu; 3 Department of Biomedical Sciences, Nazarbayev University School of Medicine, Astana 010000, Kazakhstan; nidafarooqui95@gmail.com (N.F.); zhannur.omarova@nu.edu.kz (Z.O.); srinivasa.bolla@nu.edu.kz (S.B.); 4 Department of Medicine, Aga Khan University, Karachi 74800, Pakistan; faisal.mahmood@aku.edu; 5 Brigham and Women’s Hospital, Harvard Medical School, Boston, MA 02115, USA; qak_pk@hotmail.com; 6 South City Hospital, Karachi 75400, Pakistan; haideralinaqvi9@gmail.com; 7 Medical College, Aga Khan University, Karachi 74800, Pakistan; 8 Bridge Consultants Foundation, Karachi 74500, Pakistandrsharafshah@yahoo.com (S.A.S.); 9 Neurology Department, Ziauddin University, Karachi 75000, Pakistan; ali.hassan.neurology@gmail.com

**Keywords:** HIV, HIV-associated neurocognitive disorder, neurocognitive impairment, IHDS

## Abstract

Background: In people living with HIV (PLHIV), ongoing neuronal injury has shown a correlation with elevated levels of soluble markers of immune activation, such as sCD163. Additionally, various risk factors, such as injection drug use (IDU), can independently affect immune and cognitive functions, leading to neurocognitive impairment (NCI). However, the potential sCD163-IDU-NCI axis in ART-experienced PLHIV is not clear. This study aims to determine NCI prevalence and investigate the interplay between risk factors and sCD163 in Pakistani PLHIV. Methods: For this cross-sectional study, 150 PLHIV and 30 HIV-negative people who inject drugs (PWID) were recruited using a convenience sampling strategy. NCI screening was performed using the International HIV Dementia Scale (IHDS) tool. Blood samples from PLHIV were used to perform HIV recency testing using the Asante Rapid Recency Assay, and to evaluate sCD163 levels using ELISA. Sociodemographic and clinical data were collected from medical records. Subsequently, descriptive statistics were used to summarize data variables, while comparisons (two and multiple groups) between participants with and without NCI were conducted, respectively, using the Mann–Whitney test or Kruskal–Wallis test for continuous variables, and Fisher’s exact test for categorial variables. Receiver Operating Characteristic (ROC) curve analysis was performed to assess the discriminative ability of sCD163. Logistic regression was used to identify predictors of neurocognitive impairment. Results: The majority of PLHIV had IDU as a high-risk behavior. In PLHIV, the median age was 34.5 years (IQR: 30–41), ART duration was 35 months (IQR: 17–54), and median CD4 count was 326.5 cells/µL (IQR: 116–545.5). Long-term infections (>6 months post-seroconversion; median ART duration: 35 months; median CD4 counts: 326.5 cells/μL) were noted in 83.3% of PLHIV. IHDS-based screening showed that 83.33% (all PLHIV) and 50% (PLHIV with no IDU history) scored ≤ 9 on the IHDS, suggestive of NCI. IHDS-component analysis showed the memory recall to be significantly affected in PLHIV compared to controls (median score 3.2 versus 3.7, respectively, *p* < 0.001). Regression analysis showed only long-term infection (OR: 2.99, *p* = 0.03) to be significantly associated with neurocognitive impairment. sCD163 levels were significantly lower in PLHIV with NCI (mean = 7.48 ng/mL, SD = 7.05) compared to those without NCI (mean = 14.82 ng/mL, SD = 8.23; *p* < 0.0001), with an AUC of 0.803 (95% CI: 0.72–0.88). However, after adjusting for IDU history, the regression analysis showed an odds ratio for sCD163 of 0.998 (95% CI: 0.934, 1.067, *p* = 0.957), indicating no association between sCD163 levels and NCI. Conclusion: This study reports a high prevalence of NCI in Pakistani PLHIV, and no association between sCD163 and neurocognitive impairment in PLHIV after adjustment for a history of IDU. Long-term infection and IDU were significantly linked to NCI, while only IDU was associated with lower sCD163 levels, regardless of NCI.

## 1. Introduction

Human immunodeficiency virus (HIV) is a major public health concern worldwide [1]. Persistent immune activation mediated by HIV leads to a rapid decline in immune competence [2], and along with other mechanisms, contribute to the development of neurocognitive disorders/impairments, including a range of cognitive, motor, and behavioral symptoms [3], and the most prevalent comorbidities in people living with HIV (PLHIV) [4,5]. A recent meta-analysis found an average worldwide prevalence of neurocognitive disorders to be 50%, based on estimates from Asia (52%), Europe (50%), Africa (49.5%), and the USA (50.4%) [6]. It is important to note that differences in cohort characteristics, such as age, duration of infection, and education level, may contribute to the variation in prevalence rates in individual studies [7,8].

In addition to HIV, substance use disorders, including injection drug use (IDU), represent a significant factor contributing to neurocognitive impairment (NCI) in this population [9,10]. The use of heroin and fentanyl significantly increases the risk of NCI, including deficits in affective decision-making and working memory, independent of HIV infection [11,12]. In the context of HIV, substance abuse may exacerbate neuropathology by disrupting blood–brain barrier integrity, enhancing viral replication, impairing glial cell function, and promoting neuroinflammation [9].

Ongoing neuronal injury in PLHIV has shown a correlation with elevated levels of immune activation markers, including soluble CD14 (sCD14), soluble CD163 (sCD163), and neopterin [13]. Although elevated levels of plasma sCD163, a scavenger receptor shed from activated macrophages, have been strongly associated with NCI in PLHIV [14,15], some studies have reported a non-significant association [16,17]. sCD163 was also associated with synaptodendritic damage and microglial activation in postmortem brains of PLHIV [16].

The IHDS tool has demonstrated high practical utility for NCI screening in PLHIV, especially in resource-limited settings with varying levels of education [18,19]. IHDS has a simple three-component structure (finger tapping, alternating hand sequence, and verbal memory), which makes it practical for routine clinical use.

In Pakistan, HIV exists as a concentrated epidemic and is particularly prevalent among specific high-risk groups, such as people who inject drugs (PWID; 38.4%), transgender sex workers (7.5%), and men who have sex with men (5.4%) [20,21,22,23,24]. Despite such high HIV prevalence, the data regarding the burden, associated risk factors, and biomarkers for NCI, especially in PWID, are scant. NCI screening in PLHIV is crucial as these impairments can significantly reduce the quality of life, reduce adherence to antiretroviral treatment, and increase the risk of other morbidity and mortality. Additionally, the utility of sCD163 as a potential biomarker of NCI in PLHIV needs to be assessed in various epidemiological contexts, such as geography and high-risk behaviors, like IDU. While several studies have explored the association between sCD163 and substance abuse, they did not include neurocognitive assessments of the participants [25,26]. Hence, the potential sCD163-IDU-NCI axis in ART-experienced PLHIV is not fully clear.

Therefore, this study aimed to determine an IHDS-based prevalence of neurocognitive impairment and investigate the interplay between risk factors and sCD163 (a key biological marker of inflammation).

## 2. Materials and Methods

### 2.1. Study Design, Recruitment of PLHIV, and Data Collection

This was a cross-sectional study conducted between April 2021 and January 2023. For this study, a total of 150 PLHIV were recruited from the hospitals (Aga Khan University) and community settings through a convenience sampling study. It is important to mention that patient enrollment/recruitment was seriously affected due to COVID-related restrictions, such as lockdowns, limited access to clinical settings, etc. The sociodemographic and clinical data, such as age, gender, antiretroviral therapy (ART) status and duration, risk factors, date of HIV diagnosis, CD4 count, and viral loads (wherever available), were collected from the PLHIV medical records. We also recruited 30 HIV-negative people who inject drugs (PWID) as a comparator group. Each study subject was given a unique ID (not linked to the participant’s identity), and the information was stored in password-protected Excel sheets in an anonymized manner. The selection criteria were set to exclude any subjects with brain cancers, a prior history of neurological impairment, or trauma-related brain injury. The participants’ recruitment, IHDS-based screening, and sample collection were performed only after obtaining written informed consent from each participant. The study protocol was approved by the Ethics Review Committee of the Aga Khan (2020-5386-11774, 2021-5386-19005, 2022-5386-22964, 2023-5386-26697, and 2024-5386-31533) and Ziauddin (3021220ZABC) Universities.

### 2.2. Blood Collection and HIV Recency Testing

From each PLHIV, approximately 5 mL of blood was collected in EDTA tubes using aseptic measures. The blood was transported on ice to the Aga Khan University laboratory for further processing. The blood samples were processed to separate plasma and PBMCs, which were stored at −80 °C and in liquid nitrogen, respectively, until further use.

Plasma samples were used to perform HIV recency tests using the antibody-avidity-based Asante Rapid Recency Assay (ARRA) (Sedia Biosciences, Beaverton, OR, USA) as per the manufacturer’s instructions [27,28]. ARRA, with >99% sensitivity and >98% specificity, assures established infection status and rules out any misclassification of recent from long-term infections with validated precision and reliability [29].

### 2.3. IHDS-Based Neurocognitive Impairment (NCI) Screening

In the initial step, we pilot-tested three tools: IHDS [30], CAT-rapid [31], and MoCA [32] on representative subjects. We found that due to low literacy levels (>70% of participants had no education), the participants were able to completely and correctly attempt only the IHDS test, while they failed to fully attempt the other two tests. Hence, for NCI screening, the IHDS test was selected and administered to all PLHIV participating in the study. Briefly, IHDS consists of three components: finger tapping (FT), alternating hand sequence (AHS), and 4-word recall test (4WR), with each component scored from 0 to 4 points, yielding a total possible score of 12. For the FT test, participants were instructed to tap the first two fingers of their non-dominant hand as widely and quickly as possible for 5 s, with scoring based on the number of taps completed. The AHS involved participants performing a sequence of fist-palm-cut movements as quickly as possible for 10 s, with scoring based on the number of correct sequences. For the 4WR test, participants were presented with four words for immediate recall, followed by delayed recall after 2 min, with one point awarded for each word correctly remembered. All assessments were conducted by trained healthcare professionals in a quiet environment using standardized instructions, with immediate recording of scores. The total IHDS score was calculated by summing the scores of all three components. The entire assessment took approximately 10 min to complete. In the absence of follow-up neurological screening, we used a cut-off score of ≤9 to identify the potential occurrence of neurocognitive impairment. IHDS screening was carried out in both cases (PLHIV) and controls (HIV-negative PWID). PLHIV with potential neurocognitive impairment were assessed for response to antiretroviral therapy and monitored closely for any signs of cognitive decline. If deterioration occurred, they were referred for a comprehensive neurological evaluation at the discretion of the treating physician.

### 2.4. Enzyme-Linked Immunosorbent Assay (ELISA) for sCD163

sCD163 levels were quantified from 150 PLHIV samples using an enzyme-linked immunosorbent assay (Human Cd163 Antigen, CD163 ELISA Kit, Bioassay Technology Laboratory, Birmingham, United Kingdom) following the manufacturer’s instructions. Serum sCD163 levels were measured in triplicate, with samples and standards traceable to CD163 co-analyzed in each run. Optical density was measured at 450 nm. A standard curve was generated in each run, and concentrations were interpreted using a 4-parameter logistic regression model. Individual sample concentrations were used for statistical comparisons.

### 2.5. Statistical Analysis

Descriptive statistics were calculated to summarize continuous variables as median and interquartile range (IQR; 25–75% percentile) and categorical variables as frequencies and percentages. Group comparisons for continuous variables were performed using the Mann–Whitney test (between two groups); while the Kruskal–Wallis test, with Dunn’s post hoc test applied for pairwise comparisons, was used to compare sCD163 levels among three or more groups. Similarly, group comparisons for categorical variables were performed using Fisher’s exact test. Logistic regression models were employed to identify predictors of NCI, including sCD163, incorporating demographic and clinical variables. Receiver Operating Characteristic (ROC) curve analysis, including calculation of the area under the curve (AUC), specificity, and sensitivity, was performed to assess the discriminative ability and diagnostic performance of sCD163. The optimal threshold for sCD163 was identified using Youden’s index. Statistical analyses were conducted using GraphPad Prism version 8.4.3 and JASP version 0.18.3.0. A *p* < 0.05 was considered statistically significant for all analyses.

## 3. Results

### 3.1. PLHIV Characteristics and Recency Testing

The study included 150 participants, with a median age of 34.5 years (IQR: 30–41) and a median antiretroviral treatment (ART, comprising predominantly of Dolutegravir/lamivudine/tenofovir (DTG/3TC/TDF)) duration of 35 months (IQR: 17–54). The median viral load was 124 copies/mL (IQR: 26.5–686.5), and the median CD4 count was 326.5 cells/μL (IQR: 116–545.5). The majority of participants were male (94.7%). The high-risk behavior noted in most (66.7%) participants was injection drug use, while 26% of the participants reported no associated risk factors (Table 1). Furthermore, the PLHIV had a low co-infection burden (TB: 5%; Pneumocystis Pneumonia: 1.33%; and Syphilis: 0.67%; Table 1). Finally, long-term infection, classified based on ARRA result (generally corresponding to infections >6 months post-seroconversion [29]), combined with a median ART duration of 35 months, exceeding ARRA’s mean duration of recent infection of ~180 days [29,33], and median CD4 counts of 326.5 cells/μL, consistent with immune recovery typical of sustained ART usage [33], was noted in 83.3% of individuals (Table 1).

In the control group (HIV-negative PWID), all participants were confirmed HIV-negative, had no underlying risk factors or conditions, except IDU, had a median age of 36.5 years (IQR: 33.75–40.75), and all participants were male (Table 1). The age and gender of the participants in the control group closely matched those of PLHIV (Table 1), which removes the risk of bias based on the age and gender of participants. The controls had no attributes that met the exclusion criteria previously described.

### 3.2. Prevalence of Neurocognitive Impairment Among Study Subjects

**Figure 1:**
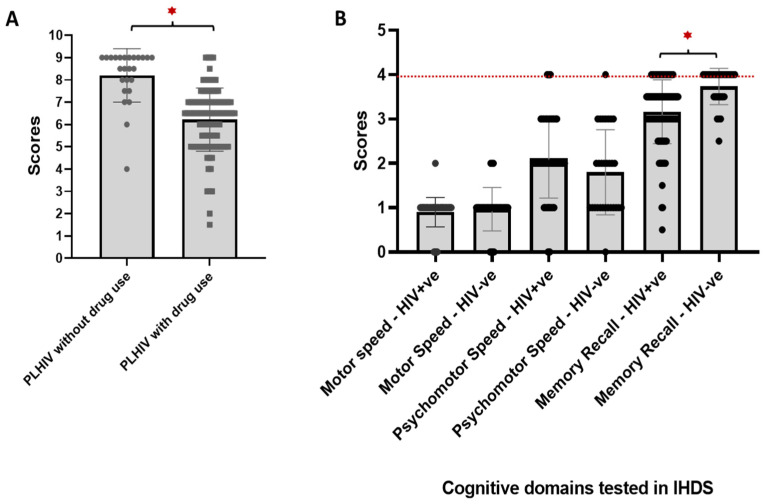
Comparative analysis of the IHDS-based cognitive domains in PLHIV and controls with neurocognitive impairment. The scatter plots display differences in (**A**) IHDS scores between PLHIVs with and without drug use history, and (**B**) the scores for three domains assessed in the IHDS test between PLHIV and controls (HIV-negative PWIDs). (**A**,**B**) * shows statistically significant differences (*p* < 0.001) between the groups. Each dot represents the individual score of each study subject, while the bar chart and error bars show the median score for each domain along with the standard deviation. (**B**) The red line indicates the maximum score achievable for each domain.

The IHDS-based screening showed that 83.33% (N = 125) of PLHIV scored ≤ 9 on the IHDS, indicating NCI. Since the majority (66.7%) of PLHIV had injection drug use as the risk factor, which could independently lead to cognitive decline [16], we carried out a subgroup analysis without including PLHIV with a history of injection drug use. The results of the subgroup analysis showed 50% (N = 25) of PLHIV scored ≤ 9 on the IHDS, indicating NCI. Comparative study of IHDS scores showed that PLHIV with injection drug use history had significantly lower IHDS scores (median: 6.5; score range 1.5–9; *p* < 0.001) as compared to IHDS without injection drug use history (median: 8.5; score range 4–9; *p* < 0.001; Figure 1A).

In the control group (HIV-negative PWID), 100% (*n* = 30) scored ≤ 9 on the IHDS, indicating NCI. Comparative analysis of the IHDS-based cognitive domains between PLHIV (cases) and the control group showed motor (cases, median: 1; control, median: 1; *p* > 0.05) and psychomotor domains (cases, median: 2.12; control, median: 1.8; *p* > 0.05) to be equally affected in the two groups. However, the PLHIV showed significantly impaired memory domain (cases, median: 3.2; control, median: 3.7; *p* < 0.001) as compared to the control group (Figure 1B).

### 3.3. Comparison of Clinical and Sociodemographic Data Among PLHIV with and Without Neurocognitive Impairment

Next, we performed a comparative analysis of the sociodemographic and clinical data of PLHIV with and without NCI. The results showed only gender, recent infection, and CD4 count to be significantly different between the two groups, where NCI group had more male participants (96%, *p* = 0.027), a lower rate of recent infection (NCI: 13.6%; no-NCI: 32.0%, *p* = 0.037), and higher CD4 counts (NCI: 418.5, IQR: 248.8–551.8; no-NCI: 212, IQR: 85.5–401; *p* = 0.023).

### 3.4. Correlates of Neurocognitive Impairment Among PLHIV

In the next step, we determined crude OR to determine the link between different variables and neurocognitive impairment. Regression analysis could only be performed for age, ART duration, and infection duration, as data in other variables were imbalanced, making them unsuitable for regression analysis. The analysis showed only long-term infection (adjusted OR: 2.99, 95% CI: 1.12–7.99, *p* = 0.03) to be significantly associated with NCI (Table 2).

### 3.5. sCD163 Levels in PLHIV with and Without Neurocognitive Impairment

**Figure 2:**
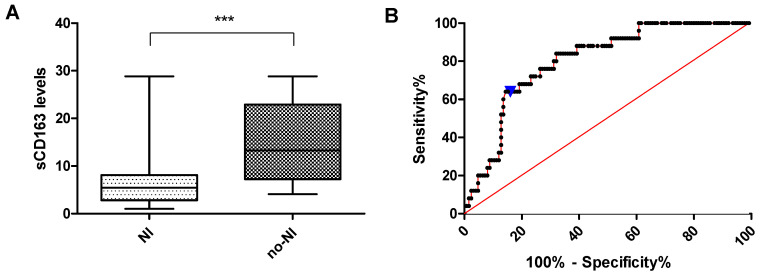
Plots comparing sCD163 levels between PLHIV with and without NCI. (**A**) The y-axis on the Box and whisker plot represents sCD163 levels (ng/mL); the x-axis denotes neurocognitive status. NI = NCI; no-NI = no NCI. *** = *p* < 0.0001. (**B**) ROC curve evaluating the diagnostic performance of sCD163 for NCI status in PLHIV. The blue inverted triangle shows the optimal threshold.

sCD163 levels differed significantly between PLHIV with and without NCI. The PLHIV with NCI (mean = 7.48 ng/mL, SD = 7.05) had significantly lower sCD163 levels compared to those without NCI (mean = 14.82 ng/mL, SD = 8.23; *p* < 0.0001) (Figure 2A). The ROC curve, with an AUC of 0.803 (95% CI: 0.72–0.88), suggests a potential discriminatory ability of sCD163 to distinguish between PLHIV with and without NCI (Figure 2). The optimal threshold of 6.60, determined using Youden’s index, provided a sensitivity of 68% and specificity of 84% (Figure 2B).

After stratifying by infection duration, PLHIV with NCI showed lower sCD163 levels (mean, recent infection = 7.44 ng/mL, SD = 6.84; mean, long-term infection = 7.48 ng/mL, SD = 7.11) compared to those without NCI (mean, recent infection = 15.44 ng/mL, SD = 7.77; mean, long-term infection = 14.52 ng/mL, SD = 8.65). This finding was independent of infection duration, as sCD163 levels did not differ significantly between recent and long-term infection, neither in the NCI group (*p* > 0.05) nor in the group without NCI.

**Figure 3:**
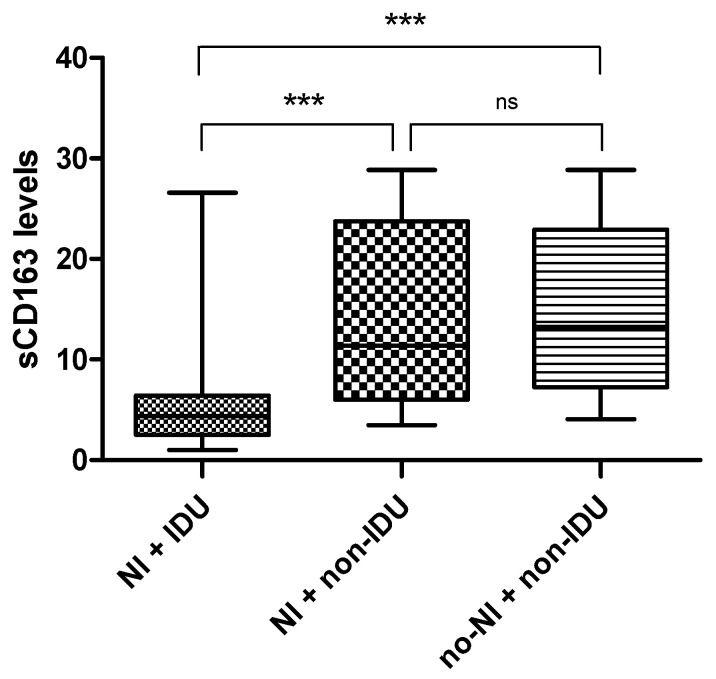
Box and whisker plot comparing sCD163 levels in PLHIV with and without NCI and no IDU history. The y-axis represents sCD163 levels; the x-axis denotes neurocognitive status and IDU history. NI + IDU = NCI with IDU history; NI + non-IDU = NCI without IDU history vs. no-NI + non-IDU = no NCI without IDU history. *** = *p* < 0.001; ns = not statistically significant.

However, after adjusting for an IDU history, sCD163 levels in PLHIV with IDU history were significantly lower (mean = 5.67 ng/mL, SD = 5.2; *p* < 0.001) than those in PLHIV without IDU history, regardless of NCI status (Figure 3). A pairwise comparison in PLHIV without a drug use history showed that sCD163 levels were comparable between PLHIV with NCI (mean = 14.69 ng/mL, SD = 8.8) and those without NCI (mean = 14.82 ng/mL, SD = 8.23, *p* > 0.05) (Figure 3).

### 3.6. Association Between sCD163 Levels, IDU History, and Neurocognitive Impairment

Logistic regression analysis was conducted (Supplementary S1) to assess the predictive value of sCD163 for NCI. In the unadjusted model, each unit increase in sCD163 levels was associated with a 10% reduction in the odds of NCI (OR = 0.90, 95% CI: 0.86–0.95, *p* < 0.001). When adjusting for age and gender, sCD163 remained a significant predictor of NCI (OR = 0.90, 95% CI: 0.85–0.95, *p* < 0.001). However, after adjusting for IDU status, sCD163 levels were not significantly associated with the increased/decreased odds of NCI (adjusted OR: 0.998;95% CI: 0.93–1.07, *p* = 0.957).

## 4. Discussion

This current study aimed to determine an IHDS-based prevalence of neurocognitive impairment and investigate the interplay between risk factors and sCD163 (a key biological marker of inflammation).

We observed a high prevalence of NCI among PLHIV. We found that long-term infection and a history of IDU were significantly linked to NCI. The prevalence of NCI observed in our study is substantially higher than that reported in Brazil (13%), Thailand (18.4%), China (34%), India (56%), Ethiopia (33.3%), Nigeria (21.5%), Malawi (14%), Botswana (38%), and Germany (6%) [8,34,35,36]. After excluding PLHIV with a history of IDU, to isolate the effect of HIV, 50% of non-IDU PLHIV scored ≤ 9 on the IHDS, indicating NCI. This prevalence is consistent with the average global prevalence of 50% [6]. This suggests that HIV infection can independently contribute to NCI. In contrast, a prior study, after accounting for heroin and fentanyl use, found no significant HIV-specific effect on cognition [11]. While previous studies have reported the prevalence of AIDS-related dementia in Pakistan to be 5% [37,38], to our knowledge, the prevalence of mild and asymptomatic NCI has not been estimated.

In our analysis, after stratifying by IDU history, PLHIV with an IDU history scored significantly lower on the IHDS test than non-IDU PLHIV. This likely underscores the additional cognitive burden imposed by IDU in PLHIV, consistent with previous studies [9,10], and requires further investigation. Chronic substance abuse itself is widely known to impair neurocognitive performance [39,40]. Several studies have shown that opioid dependence can worsen neurocognitive performance by impairing immune function and promoting viral replication [39]. Furthermore, in vitro and in vivo studies show that substances such as opioids, cocaine, and methamphetamine can stimulate viral replication, promote blood–brain barrier disruption, and activate glial cells [41]. Additionally, poly-drug use, comprising opiates (heroin and opium), marijuana/cannabis, benzodiazepines, prescription opioids, and antihistamines, reported in approximately 89.62% of PWID in Pakistan [42,43], leads to brain damage [40]. Also, poor adherence to ART in PWID may contribute to cognitive decline. The polydrug-HIV-cognitive decline axis should be further explored to elucidate the role of substance use in NCI among PLHIV.

Comparative analysis of the IHDS-based cognitive domains between PLHIV (cases) and the control group highlighted the impact of IDU on psychomotor and motor speed domains across both groups, regardless of HIV status. By contrast, memory recall deficits were more significantly observed in PLHIV, suggesting a unique effect of HIV on this aspect of cognition. Previous studies have shown that among PLHIV, working memory appears to be the most profoundly affected cognitive domain of executive function [44], which is associated with disrupted activity in frontotemporal, occipital, hippocampal, and cerebellar regions [45]. Additionally, long-term HIV infection is also associated with greater hippocampal memory decline [46]. Animal studies also show that in the hippocampus, HIV viral proteins Vpr and Tat directly impair mitochondrial transport along axons and alter the excitability and morphology of pyramidal neurons, respectively [47,48]. Taken together, these findings suggest memory systems may be particularly vulnerable in NCI. In regression analysis, only the long-term infection was significantly associated with higher odds of NCI. This can be attributed to ongoing viral replication, persistent neuroinflammation, virus- and immune activation-induced neuronal injury [49,50]. Our findings on the association between long-term infection and NCI are consistent with studies from Germany and Taiwan that found prolonged HIV infection exhibited more significant neurocognitive decline [36,51].

In the current study, PLHIV with NCI had significantly lower sCD163 levels compared with those without NCI. This contrasts with existing literature, which associates elevated sCD163 levels with NCI in PLHIV [14]. However, after stratification, this effect was attributed to a history of IDU among PLHIV, and sCD163 levels were not associated with NCI. The latter finding is consistent with a few studies that reported no association between sCD163 levels and NCI in PLHIV [16,17].

In this study, the IDU, irrespective of NCI status, was linked to significantly lower sCD163 levels in PLHIV. The link between sCD163 levels and IDU is conflicting, where some studies have shown that in both HIV-positive and HIV-negative populations, PWID typically have elevated sCD163 levels; in particular, opioid use, heroin use, and methamphetamine injection were associated with elevated sCD163 levels [25,52]. On the contrary, other studies have found no difference in sCD163 levels between PLHIV with and without opioid use disorder [53], with and without heroin use [26], or between those with and without opioid or cocaine use [54]. One possible explanation for our finding is that drug use can also reduce the number of monocytes; for example, methamphetamine administration has been shown to reduce monocyte numbers and impair their function [55], particularly phagocytosis and antigen presentation [56]. Furthermore, lower sCD163 levels may also reflect a complex interplay of cytokine- and platelet-mediated regulation associated with drug withdrawal. Acute withdrawal from substances such as heroin has been shown to induce a Th2 immune response, while elevating IL-4 and IL-10 [57]. IL-4 is a potent suppressor of CD163 expression [58,59]. Although IL-10 typically strongly induces CD163 expression, it may be insufficient to counteract inhibitory signals, such as platelet-derived CXCL4 [60], which can rise during acute methamphetamine withdrawal [61]. These mechanisms may contribute to the reduced sCD163 observed in PWID. Interestingly, IL-4, IL-10, and CXCL4 have also been shown to normalize with prolonged abstinence [57,61], suggesting that sCD163 levels may fluctuate over time; however, this remains uncertain due to limited existing literature. Therefore, there is a need for longitudinal studies to better understand the mechanisms regulating sCD163 in PWID, how its levels change over time, and its relevance in PLHIV.

While the study provides valuable insights into the prevalence and potential contributors to NCI in Pakistani PLHIV, it has the following limitations: the study relied on the IHDS tool only due to the low literacy levels of our participants. IHDS has higher practical utility in resource-limited settings with varying levels of education [18,19]. This tool has been cross-culturally validated in multiple countries, maintaining comparable sensitivity and specificity rates [18,19,31]. Although no further clinical assessment was performed, we used a lower cutoff of ≤9 rather than ≤10. For screening of NCI, a cutoff value of ≤9 has demonstrated increased specificity (79%) and lower sensitivity (49%) compared with a cutoff value of ≤10 (lower specificity (58%) and reasonable sensitivity (70%)) [19]. Another study suggested ≤9 to be used to screen neurocognitive disorder in the absence of further clinical assessments in low literacy cohorts [62]—although it risks the potential under-identification of cases [63]. Secondly, in this study, the negative controls were limited to HIV-negative PWID, excluding other risk groups. Finally, sCD163 levels could not be tested in HIV-negative PWID, as both existing kits and funding to procure additional kits were exhausted.

In conclusion, this study reveals a high prevalence of NCI among PLHIV (in Pakistan) and suggests that sCD163 may not be a reliable biomarker for NCI in PLHIV, especially those with a history of drug use, as its levels appear to be strongly influenced by a history of IDU rather than HIV-related immune activation alone. Future efforts should focus on validating IHDS screening, investigating links between IDU, long-term infection, other potential biomarkers, and NCI, and developing targeted strategies to improve neurocognitive outcomes in PLHIV. The key strategies can involve (a) early and regular screening of NCI in HIV clinics, (b) early management of NCI through cognitive, behavioral, and medical therapies, (c) improving/ensuring social (especially family) support and electronic (such as through mobile phones)/in-person reminders to reduce forgetfulness and increase ART adherence, (d) opioid-substitution therapies for IDUs, and finally (d) integrating drug rehabilitation and adherence support, for example, through residential ART adherence centers that can provide a stable environment and counseling, and further enhance compliance [64,65,66,67,68].

## Figures and Tables

**Table 1:** Demographic and clinical characteristics of PLHIV.

Variable (PLHIV)	Median (IQR 25 to 75% Percentile)
Age	34.5 (30–41)
ART duration (months)	35 (17–54)
Viral load (copies/mL)	124 (26.5–686.5)
CD4 count (cells/µL)	326.5 (116–545.5)
Variable (PLHIV)	N (%)
Gender	
Male	142 (94.7%)
Female	8 (5.3%)
Recency testing	
Recent infection	25 (16.7%)
Long-Term Infection	125 (83.3%)
Risk factors or underlying conditions	
TB	5 (3.33%)
Chronic Diarrhea	2 (1.33%)
Ischemic Heart Disease	1 (0.67%)
Pneumocystis Pneumonia	2 (1.33%)
Syphilis	1 (0.67%)
Injection Drug Use	100 (66.67%)
None	39 (26.00%)
Variable (Controls)	
Age	Median (IQR): 36.5 (33.75–40.75)
Gender (male)	N(%): 30 (100%)
HIV status (negative)	N(%): 30 (100%)
Risk factors or underlying conditions	
Injection Drug Use	N(%): 30 (100%)

**Table 2:** Factors significantly associated with NCI. The table presents the factors associated with NCI. The table shows the crude odds ratio (OR) along with 95% CI (lower-upper) and p-values, while statistically significant variables are indicated by *.

Variable	OR	95%CI (Lower-Upper)	*p*-Value
Age (years)	0.87	0.53–1.43	0.59
ART Duration (months)	0.92	0.32–2.71	0.89
Infection duration (long-term)	2.99	1.12–7.99	0.03 *

## Data Availability

All data are available in the manuscript and its Supplementary File.

## Supplementary Materials

The following supporting information can be downloaded at: https://www.mdpi.com/article/10.3390/v17091232/s1, Supplementary S1: variables used for regression analysis.
